# Synthesis of Cyclic Hexapeptides via the Hydrazide Method and Evaluation of Their Antibacterial Activities

**DOI:** 10.3390/molecules30112444

**Published:** 2025-06-03

**Authors:** Yunfei Cui, Meng Liu, Binghui Ruan, Zhouyuji Liao, Xue Tang, Dongting Zhangsun, Yong Wu, Sulan Luo

**Affiliations:** 1 Guangxi Key Laboratory of Special Biomedicine, School of Medicine, Guangxi University, Nanning 530004, China; 18878975696@163.com (Y.C.); lm315_y@163.com (M.L.); m18279871319@163.com (B.R.); 15170245109@163.com (Z.L.); 2228391035@st.gxu.edu.cn (X.T.); zhangsundt@163.com (D.Z.); 2 Key Laboratory of Tropical Biological Resources of Ministry of Education, Hainan University, Haikou 570228, China

**Keywords:** hydrazide-based chemistry, antimicrobial peptides, AMPs, stability

## Abstract

Antimicrobial peptides (AMPs) have emerged as promising candidates in the fight against multidrug-resistant pathogens due to their broad-spectrum antimicrobial activity and low potential for resistance development. However, their clinical application is limited by poor stability and susceptibility to enzymatic degradation. This study aims to address these limitations by synthesizing a series of cyclic hexapeptides using the hydrazide method and evaluating their antimicrobial activity and stability. The hydrazide method facilitated the synthesis of 11 cyclic peptides through a reaction between C-terminal hydrazides and cysteine-containing peptides. Antimicrobial assays showed that Cy-f2 and Cy-f4 exhibited potent inhibitory effects against different kinds of bacteria, including *E. coli*, *Staphylococcus aureus*, and *S. aureus*. Hemolysis assays revealed minimal red blood cell lysis at effective antimicrobial concentrations, indicating good biocompatibility. Stability tests demonstrated improved stability of the cyclic peptides compared to linear counterparts in SGF and 80 °C. In conclusion, the cyclic hexapeptides synthesized in this study demonstrate excellent antimicrobial activity, enhanced stability, and low toxicity, suggesting their potential as new candidates for treating drug-resistant bacterial infections.

## 1. Introduction

In recent decades, the frequent and incorrect use of antibiotics has caused the problem of bacterial resistance to become a public health crisis [1]. Antimicrobial peptides (AMPs) have shown unprecedented advantages as promising biomaterials against multidrug-resistant bacteria [2]. They have broad-spectrum antibacterial activity and are less likely to develop resistance, demonstrating significant potential in the fight against infection [3]. Due to their strong antimicrobial potential and unique mechanism of action, AMPs are considered an ideal candidate for antibiotic replacement [4]. Despite this potential, they are limited by instability, protease sensitivity, hemolysis, and toxicity, all limiting their clinical application [5].

To address the challenges associated with antimicrobial peptides (AMPs), researchers have developed various strategies to enhance their therapeutic potential. For instance, to extend the half-life of AMPs in the digestive tract, multiple approaches to resist proteolytic degradation have been devised [6], including rational design of amino acid sequences, combination with protease inhibitors, substitution with non-natural amino acids, design of AMP mimetics, terminal modifications, and cyclic design [7]. Among these, cyclic design stands out as particularly effective, as it introduces spatial steric hindrance that compacts the arrangement of amino acid side chains, thereby slowing proteolytic degradation and improving stability [8]. Cyclic peptides have achieved notable success in drug development, with well-known examples including antibiotics such as vancomycin [9], daptomycin [9], and polymyxin B [10]. Recently, a novel tethered macrocyclic peptide antibiotic, zosurabalpin, has been identified, demonstrating efficacy against the multidrug-resistant pathogen carbapenem-resistant Acinetobacter baumannii (CRAB) [11], thereby further highlighting the potential of cyclic peptides. Despite their clinical success or promising potential, these compounds are not without drawbacks, as their toxicity remains a concern. Additionally, cyclic peptide antibiotics used in clinical or preclinical settings often require extensive modifications, which increase synthesis costs [12]. Consequently, discovering peptides with low toxicity and minimal modifications holds significant value for clinical development.

To develop therapeutic agents with prolonged duration and enhanced stability, researchers have increasingly employed cyclization strategies for peptide modification. Over the past few decades, the chemical synthesis of cyclic peptides has seen notable progress. Several ligation strategies, such as Kent’s native chemical ligation (NCL) [13], Liu’s hydrazide-based NCL [14,15], Li’s Ser/Thr ligation [16], and Bode’s KAHA ligation [17], have been developed for synthesizing large proteins or polypeptides. These methods have expanded the possibilities for creating complex peptide structures. Among these, Liu’s hydrazide-based NCL stands out as particularly practical for cyclization [14]. This technique involves a chemically selective reaction between C-terminal hydrazides and Cys-peptides, forming native peptide bonds. It allows for easy preparation of hydrazides using Boc- or Fmoc-solid phase peptide synthesis (SPPS), a standard method in peptide chemistry. This approach has successfully synthesized toxins like mambalgin [18] and spider-venom peptide Hi1a [19], demonstrating its utility.

Based on the experimental statistics, antimicrobial peptides (AMPs) are rich in basic amino acids such as arginine (Arg, R) and lysine (Lys, K), which impart cationic properties, enabling interaction with negatively charged bacterial membranes [20]. They also contain a high proportion of hydrophobic amino acids, such as leucine (Leu, L), isoleucine (Ile, I), and phenylalanine (Phe, F), which facilitate membrane insertion and disrupt membrane integrity [21]. Their amphipathic structure allows them to remain soluble in aqueous solutions while acting effectively in lipid membrane environments, enhancing antimicrobial activity [22].

**Figure 1:**
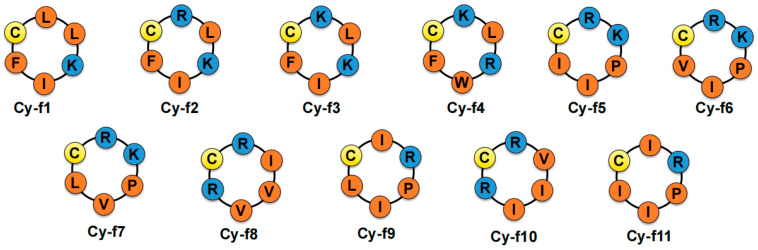
Structures of cyclic hexapeptides (Cy-f1 to Cy-f11). Each cyclic hexapeptide consists of six amino acid residues, with the specific amino acid composition indicated by single-letter codes. The single-letter codes for amino acids are as follows: C, Cys (Yellow). F, Phe (Orange), I, Ile (Orange). K, Lys (Blue). P, Phe (Orange). R, Arg (Blue). L, Leu (Orange). V, Val (Orange).

Building on this, the current study designs short peptides with antimicrobial properties. However, the cyclic nature of these peptides, due to ring strain, steric hindrance, and conformational rigidity, presents significant challenges for head-to-tail cyclization. Structurally, small rings limit the flexibility of the backbone, hindering the formation of stable secondary structures and biologically active conformations [23]. From an energetic perspective, high cyclization free energy (ΔG) and activation energy (Ea) make the reaction less favorable, while limited hydrogen bonding further destabilizes the system. Optimal cyclization is typically achieved with 6–12 amino acids to balance structural stability and thermodynamic feasibility [24]. Using the hydrazide method for peptide cyclization, this study introduced one cysteine residue along with five amino acids (mainly positively charged and hydrophobic) to synthesize a series of hexapeptides. Eleven successful cyclic peptides were synthesized (Figure 1). Antimicrobial activity testing revealed that eight peptides exhibited significant activity, with two (cy-f2 and cy-f4) showing broad-spectrum antimicrobial properties, warranting further investigation. This research provides valuable insights for future cyclic hexapeptide studies.

## 2. Results

### 2.1. Synthesis of Cyclic Hexapeptides

**Figure 2:**
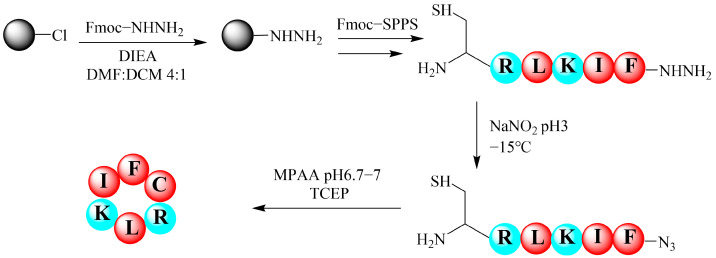
Schematic representation of the synthesis of cyclic hexapeptide Cy-f2 via the hydrazide method. The process begins with the preparation of Fmoc-hydrazide resin using 2-chlorotrityl chloride resin, followed by solid-phase peptide synthesis (SPPS) to assemble the linear hexapeptide H-Cys-Arg-Leu-Lys-Ile-Phe-NHNH_2_ (C, R, L, K, I, F denoted by colored circles). The C-terminal hydrazide is activated with NaNO_2_ at pH 3 and −15 °C to form a thioester intermediate, which undergoes intramolecular cyclization in the presence of MPAA at pH 6.7–7, with TCEP as a reducing agent, yielding the cyclic peptide Cy-f2.

**Figure 3:**
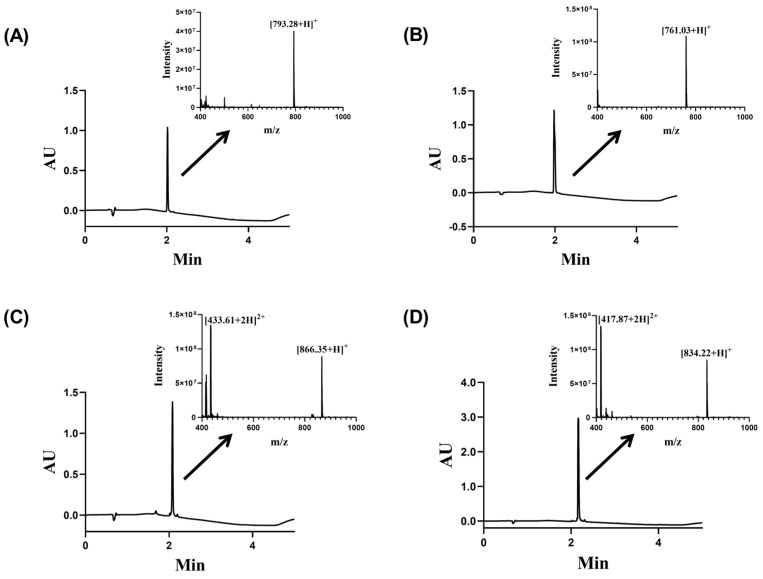
RP-UHPLC and mass spectrometry analysis of cyclic hexapeptide Cy-f2 and Cy-f4. (**A**) RP-UPLC and mass spectrum of purified linear f2-NHNH_2_. (**B**) RP-UPLC and mass spectrum of purified Cy-f2. (**C**) RP-UPLC and mass spectrum of purified linear f4-NHNH_2_. (**D**) RP-UPLC and mass spectrum of purified Cy-f4.

Peptides containing an N-terminal cysteine serve as effective precursors for the synthesis of amino acid-containing cyclic peptides. This approach relies on the in situ generation of a thioester from the C-terminal hydrazide under NaNO_2_ activation, followed by intramolecular thioester exchange to form a macrocyclic thioester, which undergoes spontaneous S-to-N acyl migration to yield the cyclic product. The synthesis of the cyclic hexapeptide Cy-f2 exemplifies this strategy (Figure 2). The linear hexapeptide hydrazide H-Cys-Arg-Leu-Lys-Ile-Phe-NHNH_2_ was first synthesized and then cyclized. The product’s purity was confirmed by chromatography, and its molecular weight was verified by ESI-MS. Chromatographic and mass spectrometric data for the key cyclic peptides Cy-f2 and Cy-f4 are presented in Figure 3. A total of 13 cyclic hexapeptides were designed and synthesized (Table S1). Their linear peptide precursors were successfully obtained, with chromatographic and mass spectrometric profiles shown in Figure S1. Of these, 11 were successfully cyclized (Table 1), with the cyclization process detailed in Figure S2 and the final cyclic products characterized in Figure S3. However, cyclization failed for the remaining two peptides (f12–f13), despite successful synthesis of their linear precursors.

### 2.2. Antimicrobial Assay of 11 Cyclic Hexapeptides

**Figure 4:**
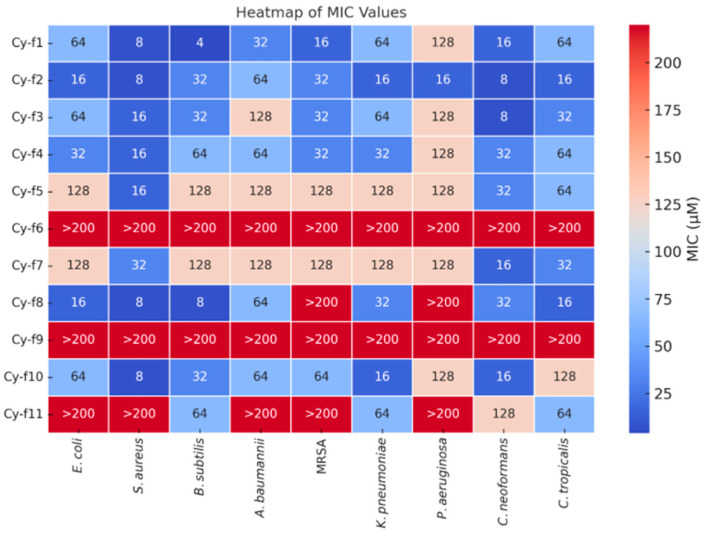
The heatmap displays the minimum inhibitory concentrations (MIC, μL) of peptides Cy-f1 to Cy-f11 against various bacterial and fungal strains (x-axis). Color intensity indicates MIC values, with blue representing lower MIC values (higher activity) and red indicating higher MIC values lower activity.

The antimicrobial and antifungal activities of 11 cyclic hexapeptides (Cy-f1 to Cy-f11) were evaluated by determining their minimum inhibitory concentrations (MICs) against a panel of pathogens (Figure 4). These pathogens included Gram-negative bacteria (*Escherichia coli*, *Klebsiella pneumoniae*, *Pseudomonas aeruginosa*), Gram-positive bacteria *(Staphylococcus aureus*, *Bacillus subtilis*, *Staphylococcus aureus* [*MRSA*], *Acinetobacter baumannii*), and fungi (*Candida tropicalis*, *Cryptococcus neoformans*). MIC values were expressed in micromolar (μM) concentrations, representing the lowest concentration required to completely inhibit visible bacterial growth after 24 h of incubation at 37 °C or fungal growth after 48 h at 30 °C. The results, presented as the mean MIC values from three independent experiments, are summarized in Figure 4. Among the tested peptides, Cy-f2 and Cy-f8 exhibited the most potent and broad-spectrum antibacterial activity. Cy-f2 showed potent activity against *E. coli* (MIC = 16 μM), *S. aureus* (MIC = 8 μM), *B. subtilis* (MIC = 32 μM), *A. baumannii* (MIC = 64 μM), *MRSA* (MIC = 32 μM), *K. pneumoniae* (MIC = 16 μM), and *P. aeruginosa* (MIC = 16 μM). Similarly, Cy-f8 displayed excellent activity against *E. coli* (MIC = 16 μM), *S. aureus* (MIC = 8 μM), *B. subtilis* (MIC = 8 μM), *A. baumannii* (MIC = 64 μM), and *K. pneumoniae* (MIC = 32 μM), though it exhibited weaker activity against *MRSA* and *P. aeruginosa* (MIC > 200 μM). Cy-f10 also demonstrated significant activity, with MIC values of 8 μM against *S. aureus* and *B. subtilis*, 64 μM against *E. coli*, *A. baumannii*, *K. pneumoniae*, and *MRSA*, and 16 μM against *C. neoformans*, though it was less effective against *P. aeruginosa* (MIC > 200 μM).

#### Comparative Analysis of Antimicrobial Peptide Efficacy

The antimicrobial screening revealed significant variability in potency among the tested peptides. Notably, Cy-f6 and Cy-f9 demonstrated markedly reduced antimicrobial efficacy, with minimum inhibitory concentrations (MICs) surpassing 200 μM across all evaluated bacterial strains, suggesting clinically insignificant activity at the administered doses. Intermediate-performance peptides exhibited selective antimicrobial profiles: Cy-f5 and Cy-f7 showed narrow-spectrum activity, with MICs ≥ 128 μM against Gram-negative (*E. coli*, *A. baumannii*, *K. pneumoniae*) and Gram-positive (*B. subtilis*, *MRSA*) pathogens. These peptides displayed intermediate potency against *S. aureus* (16 μM and 32 μM, respectively) and *P. aeruginosa* (128 μM). Two peptides demonstrated strain-specific antimicrobial characteristics: Cy-f1 exhibited: High potency against Gram-positive organisms (*S. aureus*: 8 μM; *B. subtilis*: 4 μM), moderate activity against *MRSA* (16 μM), reduced efficacy against Gram-negative strains (*E. coli* and *K. pneumoniae*: 64 μM; *P. aeruginosa*: 128 μM). Cy-f4 presented a comparable antimicrobial spectrum: 16 μM against *S. aureus,* 32–64 μM against *E. coli* and *B. subtilis,* 32 μM against *K. pneumoniae*, 128 μM against *P. aeruginosa*.

### 2.3. Hemolytic Assay

**Figure 5:**
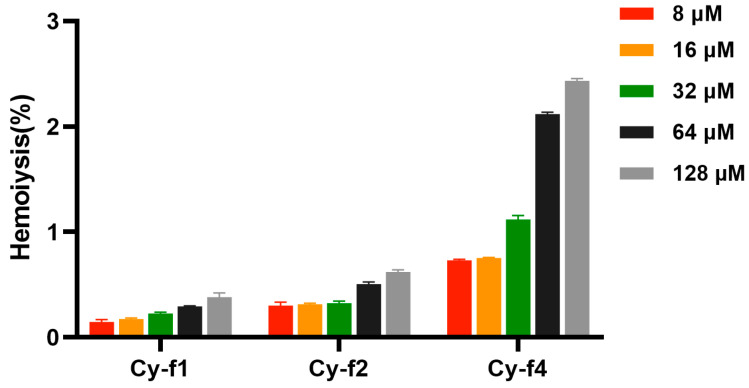
Hemolysis of Cy-f1, Cy-f2 and Cy-f4 at 8,16,32,64 and 128 μM.

Based on the antimicrobial activity screening, a hemolysis assay was conducted to assess the toxicity of Cy-f1, Cy-f2, and Cy-f4 (Figure 5). For Cy-f1, the hemolysis percentage remained consistently low across all tested concentrations. At 8 μM, Cy-f1 induced approximately 0.12% hemolysis. Notably, Cy-f1 demonstrated exceptional hemocompatibility, maintaining a low hemolysis rate of approximately 0.3% even at the elevated concentrations 64 and 128 μM, which was significantly below the threshold specified by national standards. For Cy-f2, the hemolysis percentage also remained consistently low across all tested concentrations. At 8 μM, Cy-f2 induced approximately 0.3% hemolysis, and Cy-f2 stayed stable at 0.3% at 16 μM and 32 μM. Even at higher concentrations of 64 μM and 128 μM, hemolysis levels remained minimal, reaching approximately 0.5% and 0.6%, respectively. These results indicate that Cy-f2 exhibits negligible hemolytic activity, demonstrating high biocompatibility with red blood cells at concentrations up to 128 μM. Cy-f4 exhibited a concentration-dependent increase in hemolytic activity. At 8 μM, it induced approximately 0.7% hemolysis, similar to Cy-f2 at the same concentration. However, hemolysis increased slightly with higher concentrations, reaching approximately 0.7% and 1.1% at 16 μM and 32 μM, respectively. A notable increase was observed at 64 μM, where Cy-f4 induced approximately 2.1% hemolysis, further rising to around 2.4% at 128 μM. These findings suggest that Cy-f4 exhibits mild hemolytic activity at higher concentrations. Overall, at antimicrobial effective concentrations, both Cy-f1, Cy-f2, and Cy-f4 demonstrated minimal hemolytic activity, indicating a high safety profile. In this experiment Triton X-100 was used as a positive control with a hemolysis rate of 100%. Scanning electron micrographs of red blood cells (RBCs) are presented in Figure S4. RBCs treated with Cy-f1, Cy-f2, and Cy-f4 maintained well-preserved cell morphology.

### 2.4. Time-Dependent Growth Kinetics of Cy-f2 Against Klebsiella pneumoniae

**Figure 6:**
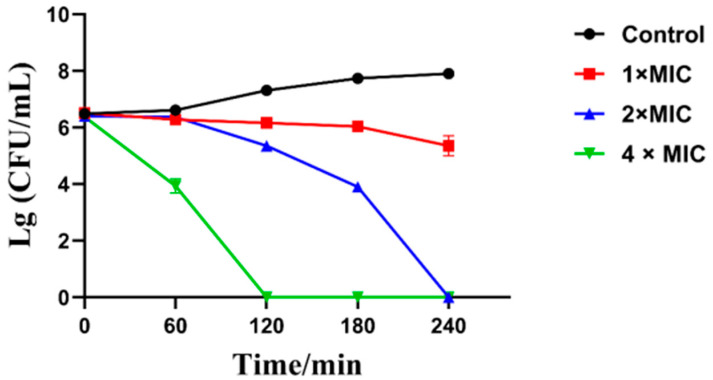
The time-dependent growth kinetics of Cy-f2 against *Klebsiella pneumoniae* at concentrations of 1 MIC, 2 MIC, and 4 MIC at 37 °C.

The time-dependent growth kinetics of the cyclic peptide Cy-f2 against *Klebsiella pneumoniae* were evaluated at concentrations of 1× MIC (16 μM), 2× MIC (32 μM), and 4× MIC (64 μM), based on the previously determined MIC value (Figure 6). Bacterial viability, expressed as logarithmic colony-forming units per milliliter (Lg CFU/mL), was monitored over 240 min at 37 °C. Results are presented in Figure 6, X as the mean Lg CFU/mL ± standard deviation (SD) from three independent experiments. At 1× MIC, Cy-f2 reduced the Lg CFU/mL from 6.55 to 5.60 after 240 min, corresponding to a 2.4 Lg CFU/mL reduction. At 2× MIC, a more pronounced effect was observed, with the Lg CFU/mL decreasing to 4.0, a 3.9 Lg CFU/mL reduction. The most significant bactericidal activity occurred at 4× MIC, where the Lg CFU/mL dropped to below 1.0 within 240 min, reflecting a >7.5 Lg CFU/mL reduction, with a rapid decline within the first 120 min. These results demonstrate that Cy-f2 exhibits concentration-dependent bactericidal activity against *K. pneumoniae*, with 4× MIC achieving near-complete bacterial eradication within 240 min. This rapid killing profile supports Cy-f2’s potential as an effective antimicrobial peptide.

### 2.5. Scanning Electron Microscopy Analysis of Cy-f2 and Cy-f4 Effects on Klebsiella pneumoniae and Candida tropicalis

**Figure 7:**
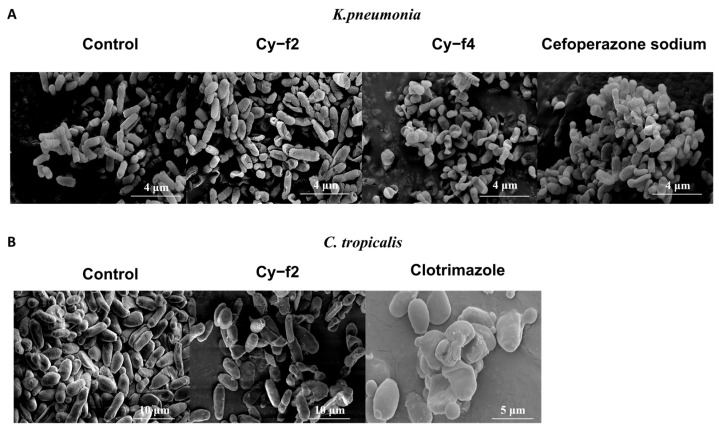
SEM images of cell morphology of (**A**) *K. pneumoniae* after treatment with control, Cy-f2, Cy-f4 and cefoperazone sodium. SEM images of cell morphology of (**B**) *C. tropicalis* after treatment with control and Cy-f2 and *Clotrimazole*.

To investigate the morphological effects of the cyclic peptides Cy-f2 and Cy-f4 on *Klebsiella pneumoniae* and *Candida tropicalis*, scanning electron microscopy (SEM) was performed (Figure 7). Microbial cells were treated with Cy-f2 or Cy-f4 at their respective 4 MIC concentrations (64 μM for Cy-f2 and 128 μM for Cy-f4 against *K. pneumoniae*; 32 μM for Cy-f2 and 128 μM for Cy-f4 against *C. tropicalis*, as determined in Figure 7) for 1 h at 37 °C for *K. pneumoniae* and 30 °C for *C. tropicalis*. Untreated cells served as controls. Samples were fixed, dehydrated, and coated with gold for SEM imaging at 5000× magnification. Results are shown in Figure 7. Effects on *Klebsiella pneumoniae* (Panel A). In the control group, *K. pneumoniae* cells exhibited a typical rod-shaped morphology with smooth, intact surfaces, indicating healthy bacterial cells. Treatment with Cy-f2 resulted in noticeable morphological changes, including surface roughening, cell shrinkage, and partial membrane disruption, suggesting that Cy-f2 compromises the integrity of the bacterial cell envelope. In contrast, *K. pneumoniae* cells treated with Cy-f4 displayed more severe damage, with extensive membrane rupture, cell deformation, and leakage of intracellular contents, indicating a stronger disruptive effect on the bacterial cell structure. Treatment with cefoperazone sodium also resulted in noticeable morphological changes, including surface roughening, cell shrinkage, and partial membrane disruption. The bactericidal efficacy of Cy-f2 and Cy-f4 against *Klebsiella pneumoniae* was verified through comparative evaluation of the treatment group and the positive control group. Effects on *Candida tropicalis* (Panel B) untreated *C. tropicalis* cells in the control group appeared as smooth, spherical to oval-shaped yeast cells with intact cell walls. After treatment with Cy-f2, the fungal cells exhibited surface irregularities, cell wall deformation, and some degree of cell collapse, indicating that Cy-f2 induces structural damage to the fungal cell wall. The effect of Cy-f4 on *C. tropicalis* was less pronounced, with cells showing mild surface roughening and slight deformation but retaining overall structural integrity compared to Cy-f2-treated cells. After the administration of clotrimazole, we observed that the bacteria also exhibited fragmentation and shrinkage, thereby confirming that Cy-f2 similarly exerts bactericidal effects.

### 2.6. Stability of Cy-f1, Cy-f2, and Cy-f4

**Figure 8:**
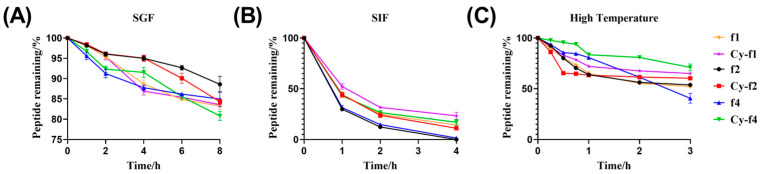
(**A**) Different time points of peptide remaining of linear f1, f2, f4 and Cy-f1, Cy-f2, Cy-f4 in SGF. (**B**) Different time points of peptide remaining of linear f1, f2, f4 and Cy-f1, Cy-f2, Cy-f4 in SIF. (**C**) Different time points of peptide remaining of linear f1, f2, f4 and Cy-f1, Cy-f2, Cy-f4 in 80 °C.

The limited stability of antimicrobial peptides (AMPs) under enzymatic conditions is a challenging hurdle to surmount for using peptides as therapeutic agents. Therefore, we chose to evaluate three aspects: simulated gastric fluid (SIF), simulated intestinal fluid (SGF), and thermal stability (Figure 8). We first synthesized the linear hexapeptides of Cy-f1, Cy-f2, and Cy-f4 using solid-phase synthesis as controls. In SGF, regardless of cyclization, the six peptides showed strong stability. After 8 h, more than 80% remains undegraded. In SIF, Cy-f1, Cy-f2, and Cy-f4 are far more stable than linear peptides. Cy-f2 and Cy-f4 remained above 20% after 2 h, while linear f2 and f4 were below 20% remaining at 2 h. At 4 h the linear f1, f2, and f4 had little remaining. The linear peptide stability of each monitoring point is worse than that of Cy-f2 and Cy-f4. At 80 °C, Cy-f4 is significantly more stable than linear peptides. The final remaining amount of cyclized f1 was kept at 65% while linear f1 was kept at 50%. The final remaining amount of cyclized f2 was kept at 60% while linear f2 was kept at 50%. This suggests that cyclization does improve peptide stability to a certain extent.

## 3. Discussion

In the present study, we designed and synthesized a series of six-amino acid cyclic antimicrobial peptides, successfully achieving their cyclization via the hydrazide method. The application of the hydrazide method not only provided an efficient and controllable approach for the synthesis of cyclic antimicrobial peptides but also significantly enhanced their chemical stability and biological activity. In this study, Cy-f2 and Cy-f4 demonstrated significant antimicrobial activity, particularly excelling in broad-spectrum antimicrobial effects, with these peptides inhibiting various Gram-positive and Gram-negative bacteria as well as fungi.

Cyclic peptides were synthesized using the hydrazide method, which involves a reaction between C-terminal hydrazides and cysteine-containing peptide segments, forming native peptide bonds, followed by ester exchange to complete the cyclization process. This method not only avoids the common complex chemical steps and low yields typically associated with traditional cyclic peptide synthesis but also improves the synthesis efficiency and purity of cyclic peptides. However, we found that the f12–f13 sequence cannot form a ring due to the rigidity and conformational constraints of these amino acids, hindering synthesis (Table S1). This is related to the specific positions and repetition of these amino acids, though the underlying pattern requires further exploration. We also observed a phenomenon where the introduction of proline into the cyclic hexapeptides led to a significant loss of activity. Proline contains an unsaturated side chain, and its cyclic structure results in lower hydrophobicity within the peptide chain. Many effective antimicrobial peptides have strong hydrophobic properties to interact with the lipid bilayer of bacterial membranes [25]. The presence of proline may reduce the hydrophobicity of the peptide, especially when its insertion position affects the hydrophobic surface of the peptide. This leads to weakened affinity between the peptide and the bacterial membrane, impairing its ability to penetrate the membrane.

**Figure 9:**
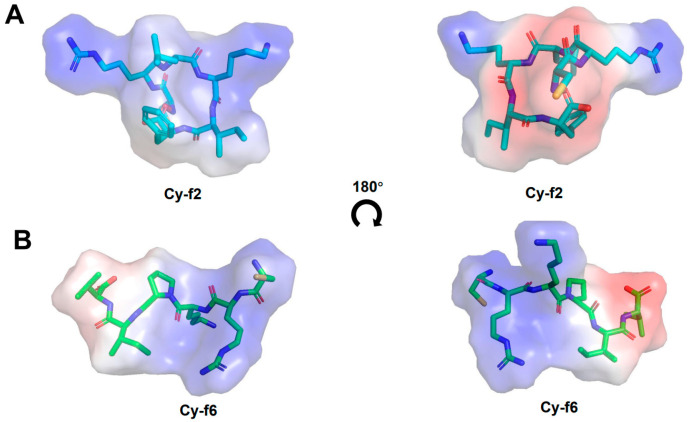
Electrostatic potential maps of cyclic hexapeptides Cy-f2 and Cy-f6. (**A**) Cy-f2, shown from two perspectives (rotated 180°). (**B**) Cy-f6, shown from two perspectives (rotated 180°). Positive electrostatic potentials are depicted in blue, and negative potentials in red.

We used alphafold2 to predict the structures of all cyclopeptides (Supplementary Materials S2). Figure 9 shows the electrostatic potential distributions of the active Cy-f2 and the inactive Cy-f4. In Cy-f2, positively charged amino acids are distributed across the peptide surface, whereas in Cy-f6, they are confined to one side. Cy-f2 contains a higher proportion of strongly hydrophobic residues (I, L, F), resulting in significantly greater overall hydrophobicity, which may enhance membrane permeability. In contrast, Cy-f6 includes the hydrophilic residue proline (P), increasing its overall hydrophilicity and potentially improving solubility in aqueous environments. Further mutational studies are needed to establish the relationship between the sequence arrangement of cyclic hexapeptides and their antimicrobial activity.

The antimicrobial mechanism of peptides is primarily through interaction with the bacterial cell membrane, disrupting membrane integrity, leading to leakage of cellular contents and ultimately causing cell death [26]. Our research results, confirmed by SEM analysis, validate this mechanism. Bacterial cells treated with Cy-f2 and Cy-f4 exhibited significant morphological changes, indicating that these peptides exert their antimicrobial effect by disrupting the membrane structure. Cy-f2 and Cy-f4 showed strong activity in broad-spectrum antimicrobial assays, including *Escherichia coli*, *Staphylococcus aureus*, *Bacillus subtilis*, and *Staphylococcus aureus* (*MRSA*). Cy-f2 was particularly notable for its antimicrobial activity, with a minimum inhibitory concentration (MIC) of 8 μM, showing strong inhibitory effects against a range of bacteria. Furthermore, time-kill kinetics experiments demonstrated that Cy-f2 exhibited concentration-dependent bactericidal activity, significantly reducing bacterial survival within a short period. Cy-f2 could serve as a template for further modification in future studies.

Hemolytic assays are an important indicator of the biocompatibility of antimicrobial peptides [27]. Our results showed that at effective antimicrobial concentrations, both Cy-f2 and Cy-f4 exhibited very low hemolysis, indicating minimal toxicity to red blood cells and good biocompatibility. Particularly for Cy-f2, even at higher concentrations, the hemolysis rate remained at an extremely low level, further proving its excellent safety. However, it is important to note that, although Cy-f2 and Cy-f4 showed low hemolytic activity in this study, as the application range of antimicrobial peptides expands, especially in clinical applications, the toxicity of peptide drugs still requires more in-depth and systematic evaluation. Future studies should integrate toxicity assessments with antimicrobial activity to optimize peptide design, ensuring both efficacy and minimal toxicity to human cells.

Stability is one of the key factors in the clinical application of antimicrobial peptides. Antimicrobial peptides typically have a short half-life in vivo and are prone to enzymatic degradation, making it crucial to improve their stability [28]. Our study demonstrated that cyclization not only enhanced the stability of the peptides in simulated gastric and intestinal fluids but also showed better tolerance under high-temperature conditions. These findings provide strong support for the clinical application of antimicrobial peptides, especially in oral administration and long-term treatment, as cyclic antimicrobial peptides may offer a longer shelf-life and better therapeutic outcomes.

While this study provides preliminary insights into the synthesis and biological properties of cyclic antimicrobial peptides, several challenges remain. First, further optimization of peptide design is needed to elucidate how amino acid sequence and cyclization strategies influence antimicrobial activity and stability. Second, comprehensive in vivo studies are required to assess toxicity and safety. Finally, advancing the clinical potential of cyclic antimicrobial peptides will depend on developing more efficient and cost-effective synthesis methods.

## 4. Materials and Methods

### 4.1. Reagents and Instruments

The reagents used in our experiments were 95–98% pure. HPLC grade solvents were employed. All the peptides were separated by using a preparative liquid chromatography system (Waters Prep 150 preparative liquid chromatography system, Milford, MA, USA). The collected solution was then filtered, diluted, and then injected into the preparative liquid chromatography column (SunFire prep C18 OBD, Waters, Milford, MA, USA) for separation. The mobile phase A is a water solution containing 0.1% TFA, and the mobile phase B is a 90% acetonitrile–water solution. The flow rate is set at 12 mL/min, and the gradient condition is set to increase mobile phase B from 5 to 70% within 40 min. The absorption wavelength is set at 214 nm. The purified sample was analyzed by Waters ACQUITY TQD tandem quadrupole mass spectrometer for molecular weight determination through ion scanning.

### 4.2. Peptides Synthesis and Characterization

#### 4.2.1. Synthesis of Cyclic Hexapeptides

All linear peptide segments (0.05 mmol scale) were synthesized on appropriate resins using standard solid-phase synthesis technique on the microwave peptide synthesizer. All Fmoc amino acids were dissolved in N,N-dimethylformamide (DMF), then were loaded on the resin with the help of N,N′-diisopropylcarbodiimide (DIC) and 2-cyano-2-(hydroxyimino) acetate (Oxyma pure) as additive to suppress racemization. After the reaction, 4-methylpiperidine in DMF (20%) was added to remove the protecting group and the next amino acid was coupled. Fmoc-hydrazine-2-chlorotrityl chloride resin was prepared through 2-chlorotrityl chloride resin as previously reported [29]. The peptide was synthesized from the Fmoc-hydrazine-2-chlorotrityl chloride resin. The peptides were then cleaved from the resin with TFA cocktail (92.5% TFA, 2.5% thioanisole, 2.5% ethanedithiol, 2.5% water). The crude peptide was precipitated with diethyl ether and lyophilized. The crude, reduced peptide was purified using preparative RP-HPLC on a C18 preparative HPLC column.

Next, we carried out the cyclization experiment with the purified peptide segment in hand. Peptide hydrazide (1 equiv.) was dissolved in the ligation buffer (0.2 M sodium phosphate acid solution containing 6 M guanidine HCl, pH 3) at the final concentration of 0.5–1 mM, after cooling to −15 °C by ice-salt bath, 36 μL of 1 M aqueous NaNO_2_ solution (10 equiv. of the first peptide segment) was added and gently stirred for 15–20 min to obtain peptide hydrazide. After that, 40 equiv. 4-carboxybenzenethio (MPAA) was added to transfer peptide hydrazide into the corresponding thioester 10 in vivo at room temperature. Then, the pH was adjusted to 6.3–6.5 for peptide cyclization known as an intramolecular N to S acyl shift reaction. After completing the above steps, we need to set different time points of 0 h, 30 min, and 2 h, then use Analytical RP-HPLC was used to monitor the process. After completion, the reaction buffer was reduced by 0.1 M tris(2-carboxyethyl)phosphine (TCEP). The product was purified by semi-preparative HPLC and lyophilized. The cyclization product peptide was confirmed by mass spectrometry.

#### 4.2.2. Peptide Molecular Weight Identification

Linear and cyclic peptides were analyzed by tandem quadrupole mass spectrometer for molecular weight determination through ion scanning. The mobile phase A was a 0.05% formic acid aqueous solution, and the mobile phase B was a 100% acetonitrile solution. The parameters were set as follows: cone voltage 45 V, cone gas flow rate 1000 L/h, capillary voltage 1.2 kV, and scan range 400–1000 Da. Every sample was completed within 7 min.

### 4.3. Biological Studies

#### 4.3.1. Antibacterial Assay

The antibacterial activity of peptide analogs was tested against different bacterial strains, all purified cyclotides underwent antibacterial testing against Gram-positive bacterium *Staphylococcus aureus* (BNCC 186335), *Bacillus subtilis* (BNCC 109047), and *Staphylococcus aureus* (MRSA, BNCC 330041), Gram-negative bacteria, including *Acinetobacter baumannii* (BNCC 337173), *Pseudomonas aeruginosa* (BNCC 125486), and *Cryptococcus neoformans* (BNCC 225501). Fungi included *Candida parapsilosis* (BNCC 336515) and *Candida tropicalis* (BNCC 340288). All the above strains are from the Henan Provincial Engineering Research Center of Industrial Microorganisms (Henan, China). *Escherichia coli* (ATCC 25922) and *Klebsiella pneumoniae* (CMCC(B) 46117) were sourced from Guangzhou HuanKai Microbial Technology Co., Ltd. (Guangzhou, China). The peptides’ MIC was determined using the Clinical and Laboratory Standards Institute broth dilution procedure with slight modifications [30,31].

Colonies of these bacterial strains were grown in respective agar media, then inoculated in Mueller Hinton (MH) broth and incubated at 37 °C overnight. The peptide solutions were first diluted 1 in 100 (from the stock solution at 20 mM) and then further two-fold dilution in MH broth in a sterile 96-well plate was performed, resulting in 100 µL of broth containing increasing concentrations. Bacteria in their exponential growth phase were diluted in MH broth, and 100 µL of this suspension was added in each well. This resulted in a final 200 µL suspension containing 0.5−1.0 × 10^6^ CFU/mL. Then, these plates were incubated at 37 °C for 20–22 h.

*Candida tropicalis* was cultured on Sabourad Dextrose Agar (SDA). A single colony was inoculated in YM broth medium and grown overnight at 37 °C while shaking. Peptides were diluted in SDB media by two-fold dilution, resulting in 100 µL of medium containing increasing concentrations. The overnight culture (turbidity at OD600 = 1) was 1000× diluted in SDB. Then 100 µL of this suspension was added to each well, resulting in a final 200 µL suspension containing 2–4 × 10^5^ CFU/mL. The plate was incubated at 37 °C for 24 h.

Each test was performed at least three times. MIC (minimum inhibitory concentration) values were determined as the concentrations of peptides that caused >99% inhibition of bacterial growth.

#### 4.3.2. Hemolytic Assay

For the hemolytic assay, 2 mL of fresh blood was collected from a healthy mouse into an EDTA-coated tube. All procedures regarding the care and use of laboratory animals adhered to the guidelines established by the Guangxi University Ethics Committee (Approval No.: GXU-2024-063). According to the method of Tan [32] with necessary adjustments. The blood was centrifuged and upper supernatant plasma was removed. The cell pellets were washed with sterile PBS three times. The washed blood cells were then diluted 25 times to make 4% concentration of the initial blood cells. Red blood cells (500 µL) were treated with increasing concentrations of analogs, with triton X-100 at 0.1% being used as positive control giving 100% hemolysis. After 1 h of incubation at 37 °C, tubes were centrifuged at 800 rpm for 5 min to pellet down red blood cells. After centrifugation, 200 µL of supernatant were transferred into a 96-well plate and the absorbance was recorded at 576 nm using a microplate reader. The hemolysis experiment of Cy-f1, Cy-f2, and Cy-f4 were repeated three times. Then, the following formula was applied to calculate percent hemolysis:

% Hemolysis = (O.D of test sample − O.D of PBS control) ÷ (O.D of Tritox × positive control − O.D of PBS control) × 100%

#### 4.3.3. Time-Dependent Growth Kinetics

*Klebsiella pneumoniae* was cultured in fresh LB nutrient broth at 37 °C following the protocol described by Huang et al. [33,34], with modifications. Upon reaching the logarithmic growth phase, the bacterial suspension was adjusted to approximately 10^6^ CFU/mL and subsequently treated with cyclized f2 at concentrations corresponding to 1×, 2×, and 4× the minimum inhibitory concentration (MIC). Aliquots were collected at 1, 2, 3, and 4 h post-treatment, serially diluted, and plated onto solid agar medium for 24 h incubation. Colony-forming units (CFUs) were enumerated to assess bacterial viability. All experiments were performed in triplicate. Time-dependent growth kinetics were analyzed by plotting bacterial survival (log_10_ CFU/mL) against time (h) using GraphPad Prism9.5, enabling statistical evaluation of the bactericidal and bacteriostatic effects of the cyclic hexapeptide.

#### 4.3.4. Scanning Electron Microscope (SEM)

According to the method of He [35] with adjustments, bacteria were grown to the logarithmic phase and then washed and resuspended in sterile PBS to an OD600 of 0.4. They were incubated with an equal volume of AMPs at 4× MIC for 1 h. After incubation, the bacterial pellets were washed three times with PBS and centrifuged at 3500 rpm for 4 min. The pellets were then fixed overnight in 2.5% (*w*/*v*) glutaraldehyde. The bacterial samples were dehydrated using a series of ethanol solutions with increasing concentrations (30%, 50%, 70%, 90%, and 100%). After dehydration, the samples were dried and sputter-coated with gold. Bacterial fragmentation was observed using scanning electron microscopy (HITACHI SU5000, Tokyo, Japan). Untreated bacterial suspensions served as the negative control.

### 4.4. Stability

#### 4.4.1. Simulated Gastric Fluid Stability

A total of 20 mg of NaCl and 30 mg of pepsin were dissolved in 70 μL of hydrochloric acid, and then the solution was diluted to 10 mL with distilled water to obtain a simulated gastric fluid (SGF) solution. The final pH of this solution was approximately 1.2. The linear peptides and their cyclized analogue lyophilized powder were dissolved and diluted to a final concentration of 0.25 mM. A SGF solution without added peptide was also set up as a blank control group. All experimental groups and control groups were set at 37 °C. A 40 μL sample was taken at the time points of 0, 1, 2, 4, 6 h and 8 h, then was reduced by 40 μL of 0.2 M Na_2_CO_3_ solution [36]. Finally, Analytical RP-HPLC was used to monitor the process, and the stability of the peptides was assessed by determining the peak area of the resulting peptides at 214 nm at each time point by calculating the percentage with the peak area at the starting time point (0 h). Each test was performed three times to ensure accuracy.

#### 4.4.2. Simulated Intestinal Fluid Stability

Dissolve 68 mg of potassium dihydrogen phosphate (KH_2_PO_4_) and 100 mg of trypsin in distilled water, followed by adjusting the pH to 6.8 using 0.2 M NaOH solution, and finally, the volume of the solution was fixed to 10 mL. The solution obtained in this step was simulated intestinal fluid (SIF). The linear peptides and their cyclized analogue lyophilized powder were dissolved and diluted to a final concentration of 0.25 mM. A SIF solution without added peptide was also set up as a blank control group. All experimental groups and control groups were set at 37 °C. A 40 μL sample was taken at the time points of 0, 1, 2, 4, 6 h, and 8 h, and immediately 40 μL of 4% TFA was added to stop the reaction [37,38]. Finally, Analytical RP-HPLC was used to monitor the process, and the stability of the peptides was assessed by determining the peak area of the resulting peptides at 214 nm at each time point by calculating the percentage with the peak area at the starting time point (0 h). Each test was performed three times to ensure accuracy.

#### 4.4.3. Thermal Stability

Peptide samples dissolved in distilled water. At the same time, a tube containing only the same volume of distilled water was set up as a blank control group for the experiment. Incubate them in an 80 °C water bath. We set different time points of 0 h, 15 min, 30 min, 45 min, 1 h, 2 h, and 3 h. For samples collected at each time point, the percentage stability of the peptide at different time points was calculated by measuring its peptide peak area at 214 nm and comparing it to the peak area at the beginning of the experiment (0 h), each test was performed three times to ensure accuracy.

Stability was expressed as the percentage recovery which was calculated from the following equation:

Recovery (%) = purity of analyzed sample ÷ purity of analyzed control (0 h) × 100%.

### 4.5. Structural Prediction by AlphaFold2

To investigate the three-dimensional structures of the synthesized cyclic hexapeptides Cy-f1 to Cy-f11, we utilized a linear peptide model for structural prediction. The structures were predicted with high accuracy using AlphaFold2 (https://colab.research.google.com/github/sokrypton/ColabFold/blob/main/AlphaFold2.ipynb, accessed on 29 May 2025), based on the amino acid sequences of the peptides [39,40]. Subsequently, the electrostatic potential of each peptide was calculated and visualized using the Advanced Poisson-Boltzmann Solver plugin in PyMOL (Version 2.5.2) [41]. In the resulting visualizations, positive electrostatic potentials were represented in blue, while negative potentials were depicted in red.

## 5. Conclusions

This study validates the effectiveness of the hydrazide-based ligation method for the synthesis of cyclic hexapeptides. Cyclization improved peptide stability to some extent, and certain cyclic hexapeptides exhibited promising antimicrobial activity, partially overcoming key limitations of antimicrobial peptides (AMPs). Among the 13 designed sequences, 11 were successfully cyclized, with Cy-f2 and Cy-f4 demonstrating broad-spectrum antibacterial efficacy, particularly against multidrug-resistant pathogens such as *Escherichia coli*, *Staphylococcus aureus*, and *MRSA* (minimum MIC of 8 μM). Microscopy confirmed their rapid bactericidal action through membrane disruption, while their low hemolysis (<2.4% at 128 μM) indicates good biocompatibility. Cyclization significantly enhanced peptide stability, with Cy-f2 and Cy-f4 outperforming their linear counterparts in simulated intestinal fluid and at 80 °C. Additionally, the incorporation of proline reduced hydrophobicity, leading to decreased antimicrobial activity in cyclic peptides. Overall, Cy-f2 and Cy-f4 represent promising candidates for combating drug-resistant infections and warrant further investigation. Future studies should focus on optimizing Cy-f2 and Cy-f4 to enhance antimicrobial activity and conducting in vivo evaluations to facilitate their clinical development.

## Figures and Tables

**Table 1:** Molecular weights of cyclic hexapeptides (Cy-f1 to Cy-f11).

Peptide Name	Sequence	Theoretical M.W. (g/mol)	Experimental M.W. (g/mol)
Cy-f1	*c*-[CLLKIF]	717.98	717.97
Cy-f2	*c*-[CRLKIF]	761.01	761.03
Cy-f3	*c*-[CKLKIF]	733.00	733.22
Cy-f4	*c*-[CKLRWF]	834.06	834.22
Cy-f5	*c*-[CRKPII]	710.95	711.35
Cy-f6	*c*-[CRKPIV]	696.92	697.25
Cy-f7	*c*-[CRKPVL]	696.92	697.53
Cy-f8	*c*-[CRIVVR]	726.95	727.34
Cy-f9	*c*-[CIRPIL]	695.94	696.75
Cy-f10	*c*-[CRVIIR]	740.98	741.27
Cy-f11	*c*-[CIRPII]	695.94	696.13

## Data Availability

Data are contained within the article and Supplementary Materials.

## Supplementary Materials

The following supporting information can be downloaded at: https://www.mdpi.com/article/10.3390/molecules30112444/s1, Table S1: Sequences and molecular weights of synthesized linear hexapeptide hydrazides(f1 to f13); Figure S1: UPLC and ESI-MS analysis of the 13 linear hexapeptide hydrazides; Figure S2: UPLC chromatogram monitoring the cyclization process; Figure S3: Chromatographic and mass spectrometric identification profiles of the synthesized cyclic hexapeptides; Figure S4: The SEM images of RBC’s treated with Cy-f1, Cy-f2, and Cy-f4. Supplementary Material S2: The electrostatic potential distributions of all cyclopeptides.
