# Supplementary material for: Synthesis of Cyclic Hexapeptides via the Hydrazide Method and Evaluation of Their Antibacterial Activities
Source: Molecules. 2025 Jun 3;30(11):2444. doi: 10.3390/molecules30112444 (PMC12156185; doi:10.3390/molecules30112444)
Supplement: Supplementary file 1 [file molecules-30-02444-s001.zip › Supppt infomation 1.pdf]

Supplementary Materials

Synthesis of cyclic hexapeptides via the Hydrazide Method and Evaluation of their Antibacterial Activities

Yunfei Cui [1], Meng Liu [1] , Binghui Ruan [1] , Zhouyuji Liao[1] , Xue Tang [1] , Dongting Zhangsun[1,2] ,Yong Wu [1,] and Sulan Luo [1,2,*]*

1 Guangxi Key Laboratory of Special Biomedicine, School of Medicine, Guangxi University, Nanning, China

2 Key Laboratory of Tropical Biological Resources of Ministry of Education, Hainan University, Haikou, China

* Correspondence: wuyong@gxu.edu.cn (Y.W.); sulan2021@gxu.edu.cn (S.L.)

Supplementary materials contain:

Table S1. Sequences and molecular weights of synthesized linear hexapeptide hydrazides(f1 to f13).

Figure S1. UPLC and ESI-MS analysis of the 13 linear hexapeptide hydrazides.

Figure S2. UPLC chromatogram monitoring the cyclization process.

Figure S3. Chromatographic and mass spectrometric identification profiles of the synthesized cyclic hexapeptides.

Figure S4.The SEM images of RBC’s treated with Cy-f1, Cy-f2, and Cy-f4.

Table S1 Sequences and molecular weights of synthesized linear hexapeptide hydrazides(f1 to f13).

**Peptide name**	**Sequence**	**Theoretical M.W.(g/mol)**	**Experimental M.W.(g/mol)**
f1	CLLKIF-$NHNH_2$	749.98	750.23
f2	CRLKIF-$NHNH_2$	793.01	793.28
f3	CKLKIF-$NHNH_2$	765.00	765.35
f4	CKLRWF-$NHNH_2$	866.06	866.35
f5	CRKPII-$NHNH_2$	742.95	743.58
f6	CRKPIV-$NHNH_2$	728.92	729.30
f7	CRKPVL-$NHNH_2$	728.92	729.30
f8	CRIVVR-$NHNH_2$	758.95	759.26
f9	CIRPIL-$NHNH_2$	727.94	728.18
f10	CRVIIR-$NHNH_2$	772.98	773.33
f11	CIRPII-$NHNH_2$	727.94	728.23
f12	CRRLVK-$NHNH_2$	787.99	787.87
f13	CRIVIR-$NHNH_2$	772.98	773.13

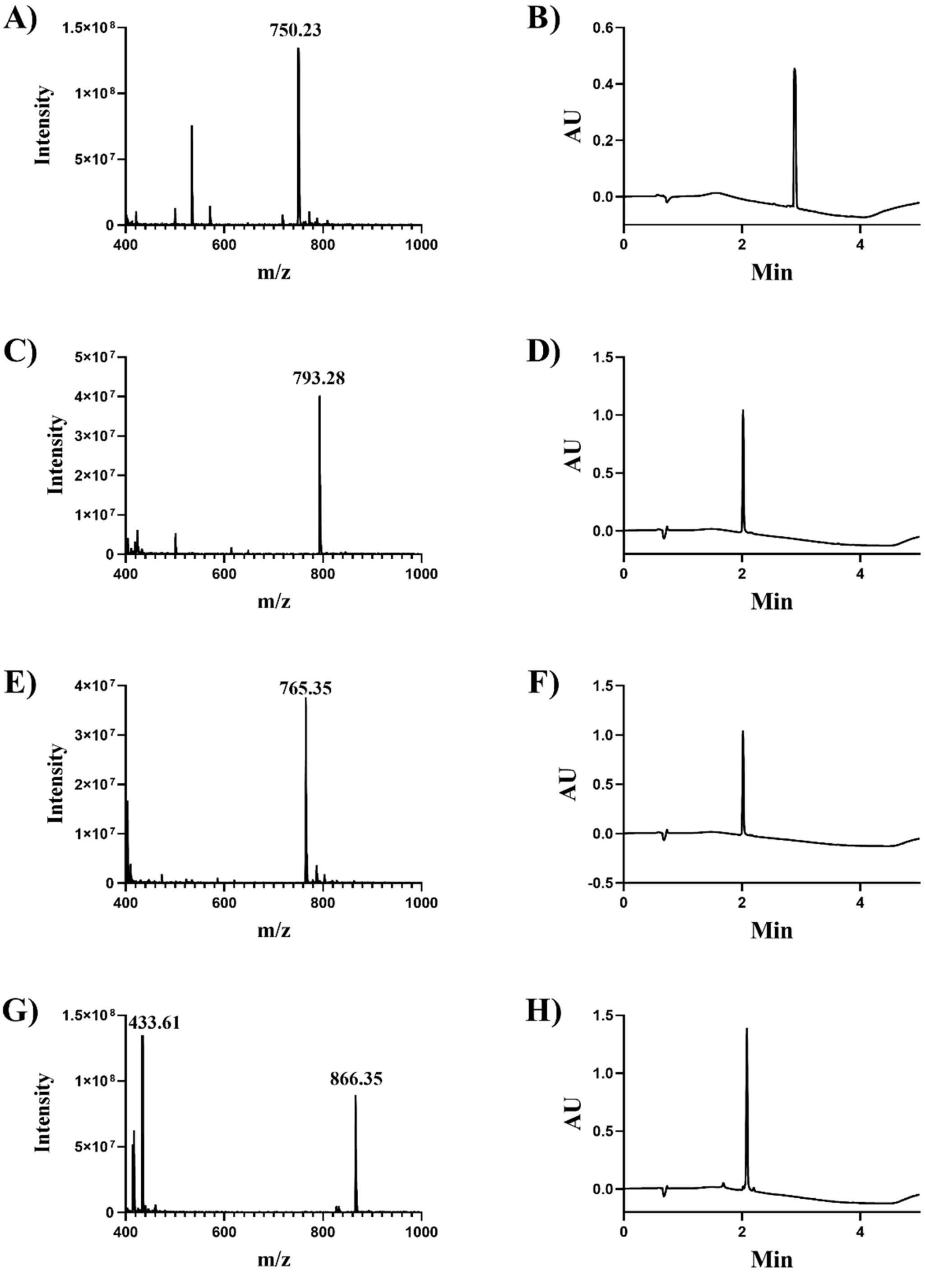
A)
1.5×10⁸
1×10⁸
5×10⁷
0
Intensity
750.23
400
600
800
1000
m/z
B)
0.6
0.4
0.2
0.0
AU
0
2
4
Min
C)
5×10⁷
4×10⁷
3×10⁷
2×10⁷
1×10⁷
0
Intensity
793.28
400
600
800
1000
m/z
D)
1.5
1.0
0.5
0.0
AU
0
2
4
Min
E)
4×10⁷
3×10⁷
2×10⁷
1×10⁷
0
Intensity
765.35
400
600
800
1000
m/z
F)
1.5
1.0
0.5
0.0
-0.5
AU
0
2
4
Min
G)
1.5×10⁸
1×10⁸
5×10⁷
0
Intensity
433.61
866.35
400
600
800
1000
m/z
H)
1.5
1.0
0.5
0.0
AU
0
2
4
Min

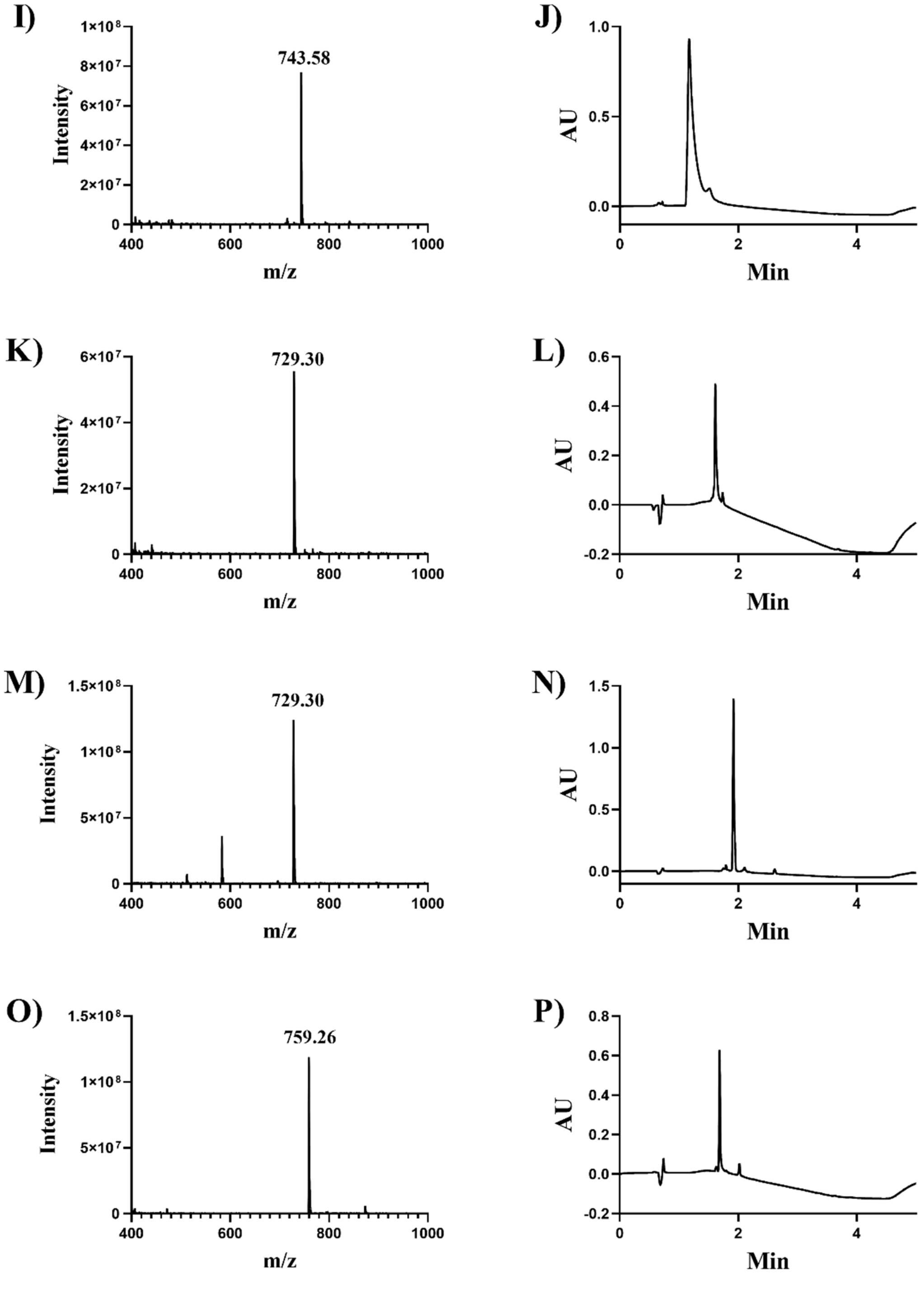

I)
Intensity
m/z
743.58
J)
AU
Min
K)
Intensity
m/z
729.30
L)
AU
Min
M)
Intensity
m/z
729.30
N)
AU
Min
O)
Intensity
m/z
759.26
P)
AU
Min

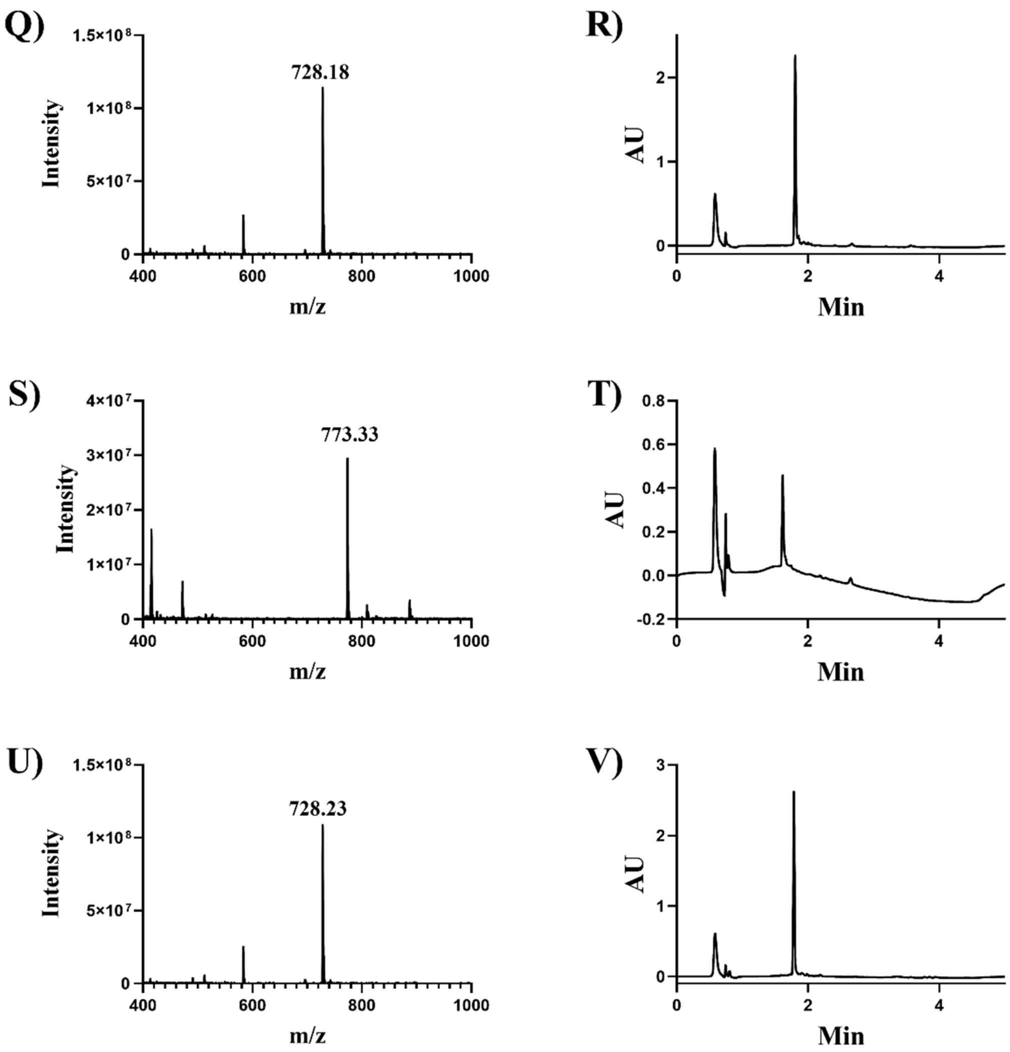
Q)
1.5×10⁸
1×10⁸
5×10⁷
0
Intensity
728.18
400
600
800
1000
m/z
R)
AU
2
1
0
0
2
4
Min
S)
4×10⁷
3×10⁷
2×10⁷
1×10⁷
0
Intensity
773.33
400
600
800
1000
m/z
T)
0.8
0.6
0.4
0.2
0.0
-0.2
AU
0
2
4
Min
U)
1.5×10⁸
1×10⁸
5×10⁷
0
Intensity
728.23
400
600
800
1000
m/z
V)
3
2
1
0
AU
0
2
4
Min

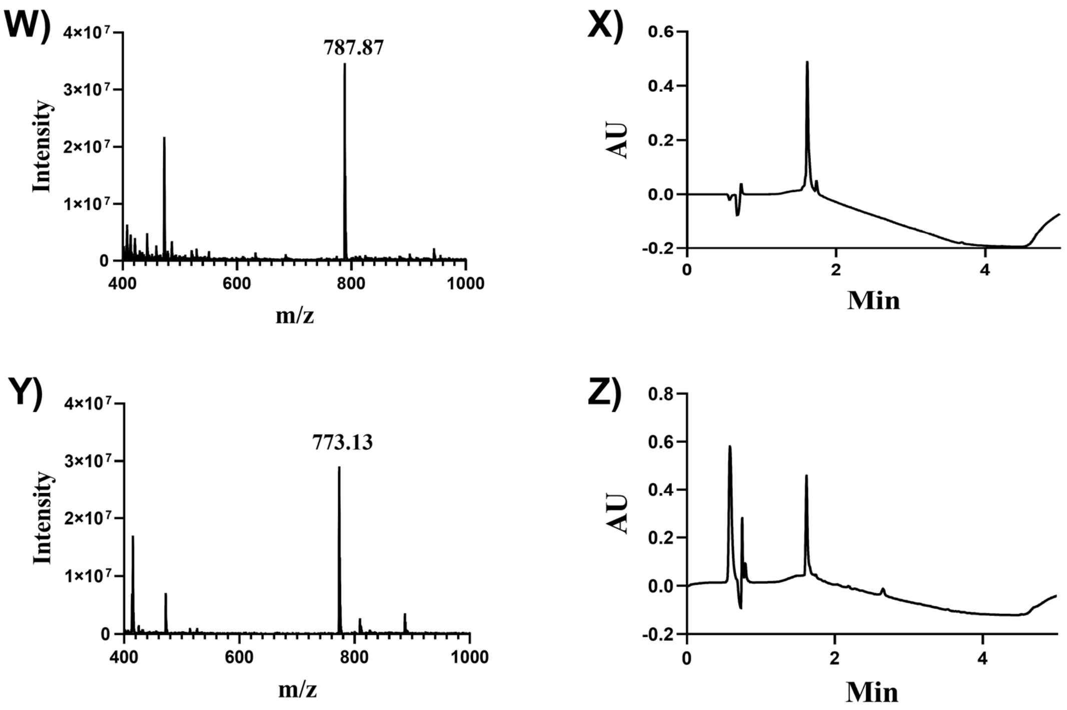

Figure S1 UPLC and Mass spectra of the series of linear hexapeptide hydrazides (UV detection at 214 nm). **(A)** The mass spectra of f1. **(B)** The UPLC profile of f1. **(C)** The mass spectra of f2. **(D)** The UPLC profile of f2. **(E)** The mass spectra of f3. **(F)** The UPLC profile of f3. **(G)** The mass spectra of f4. **(H)** The UPLC profile of f4. **(I)** The mass spectra of f5. **(J)** The UPLC profile of f5. **(K)** The mass spectra of f6. **(L)** The UPLC profile of f6. **(M)** The mass spectra of f7. **(N)** The UPLC profile of f7. **(O)** The mass spectra of f8. **(P)** The UPLC profile of f8. **(Q)** The mass spectra of f9. **(R)** The UPLC profile of f9. **(S)** The mass spectra of f10. **(T)** The UPLC profile of f10. **(U)** The mass spectra of f11. **(V)** The UPLC profile of f11. **(W)** The mass spectra of f12. **(X)** The UPLC profile of f12. **(Y)** The mass spectra of f13. **(Z)** The UPLC profile of f13.

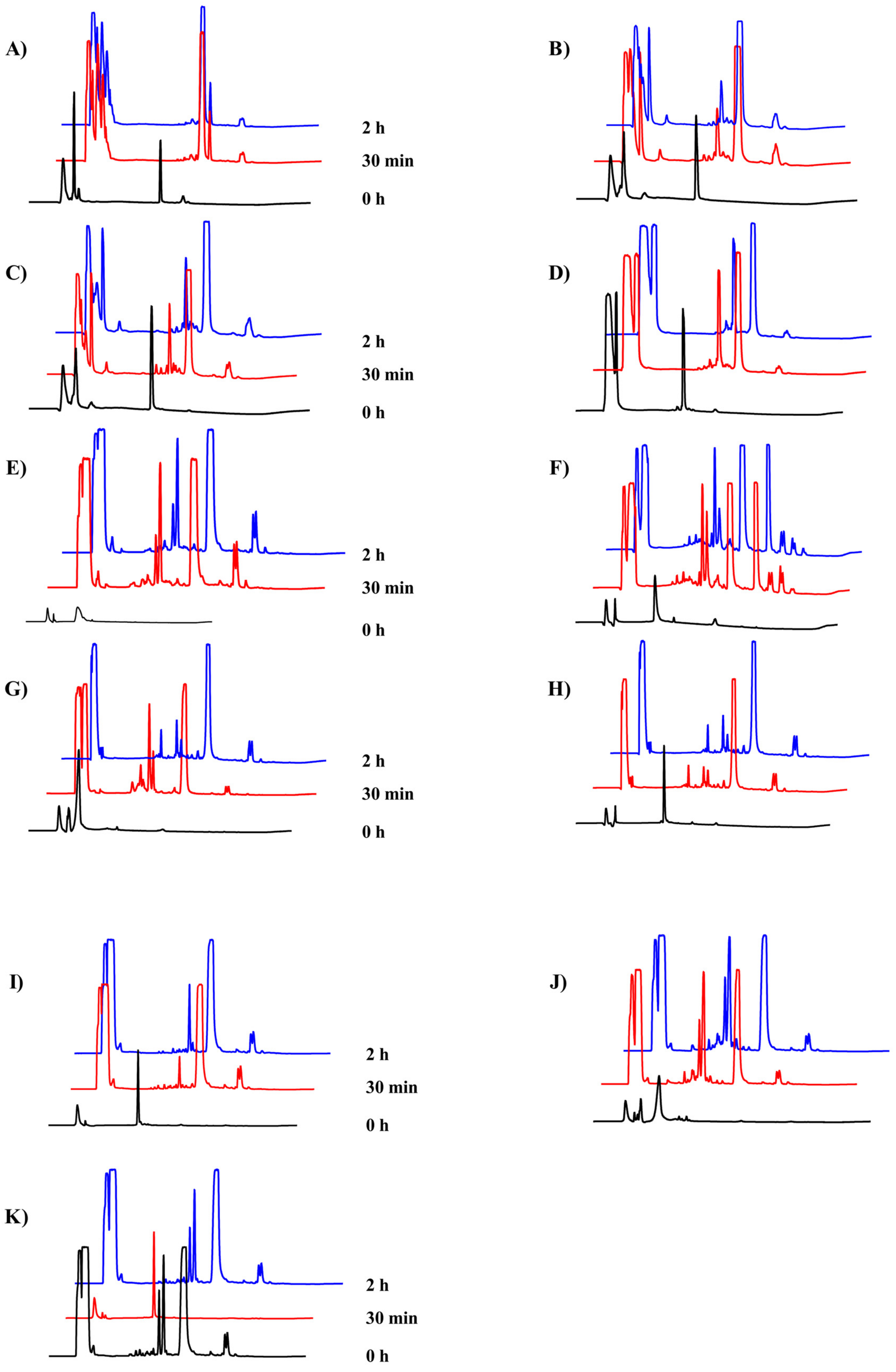

Figure S2 UPLC of the monitoring of antimicrobial peptides during cyclization (UV detection at 214 nm). **(A)** Cyclization monitoring peaks for Cy-f1 in 0 h、30 min、2 h. **(B)** Cyclization monitoring peaks for Cy-

f2 in 0 h、30 min、2 h. **(C)** Cyclization monitoring peaks for Cy-f3 in 0 h、30 min、2 h. **(D)** Cyclization monitoring peaks for Cy-f4 in 0 h、30 min、2 h. **(E)** Cyclization monitoring peaks for Cy-f5 in 0 h、30 min、2 h. **(F)** Cyclization monitoring chart for Cy-f6 in 0 h、30 min、2 h. **(G)** Cyclization monitoring peaks for Cy-f7 in 0 h、30 min、2 h. **(H)** Cyclization monitoring peaks for Cy-f8 in 0 h、30 min、2 h. **(I)** Cyclization monitoring peaks for Cy-f9 in 0 h、30 min、2 h. **(J)** Cyclization monitoring peaks for Cy-f10 in 0 h、30 min、2 h. **(K)** Cyclization monitoring peaks for Cy-f11 in 0 h、30 min、2 h.

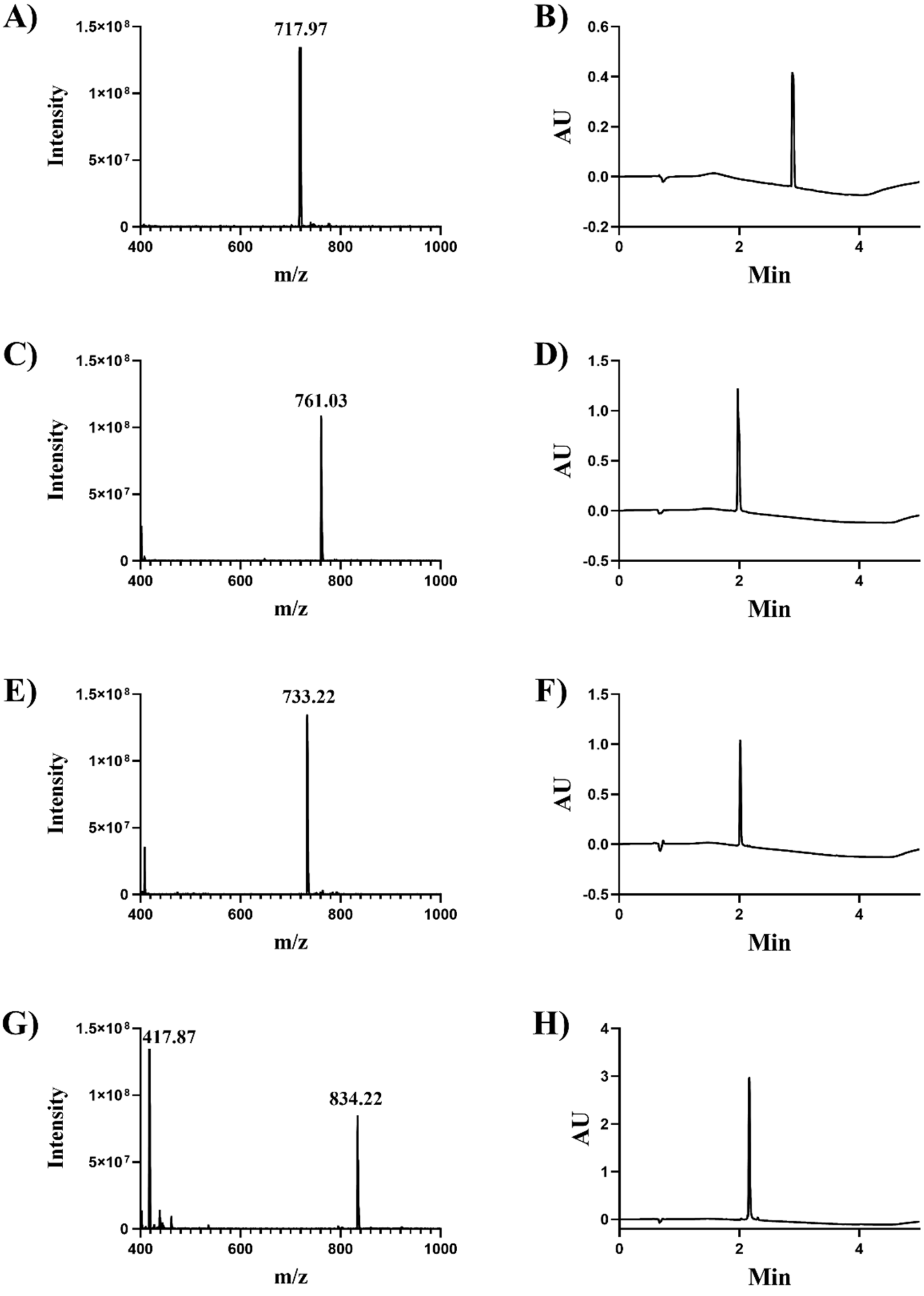

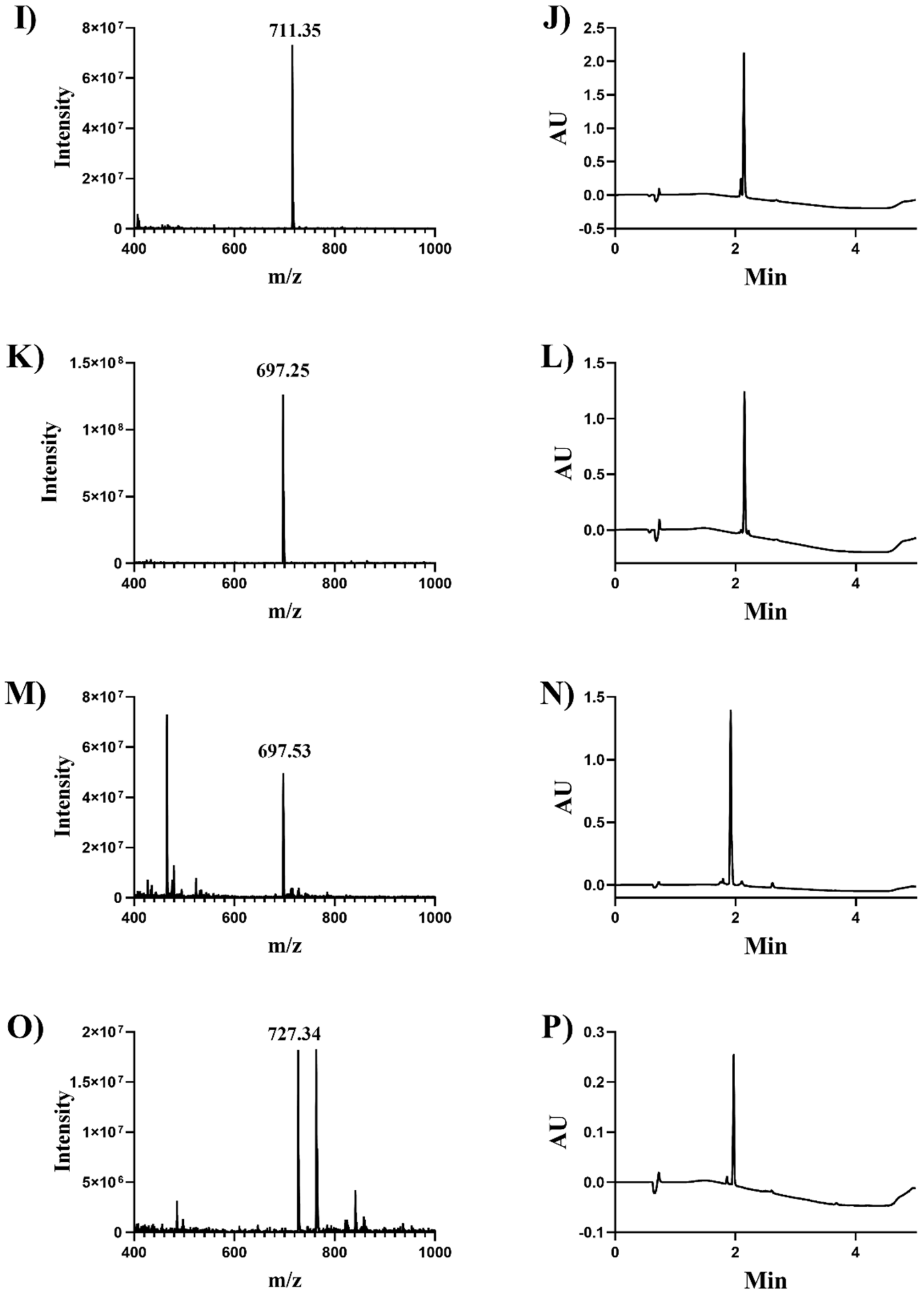
I)
711.35
Intensity
8×10⁷
6×10⁷
4×10⁷
2×10⁷
0
400
600
800
1000
m/z
J)
AU
2.5
2.0
1.5
1.0
0.5
0.0
-0.5
0
2
4
Min
K)
697.25
Intensity
1.5×10⁸
1×10⁸
5×10⁷
0
400
600
800
1000
m/z
L)
AU
1.5
1.0
0.5
0.0
0
2
4
Min
M)
697.53
Intensity
8×10⁷
6×10⁷
4×10⁷
2×10⁷
0
400
600
800
1000
m/z
N)
AU
1.5
1.0
0.5
0.0
0
2
4
Min
O)
727.34
Intensity
2×10⁷
1.5×10⁷
1×10⁷
5×10⁶
0
400
600
800
1000
m/z
P)
AU
0.3
0.2
0.1
0.0
-0.1
0
2
4
Min

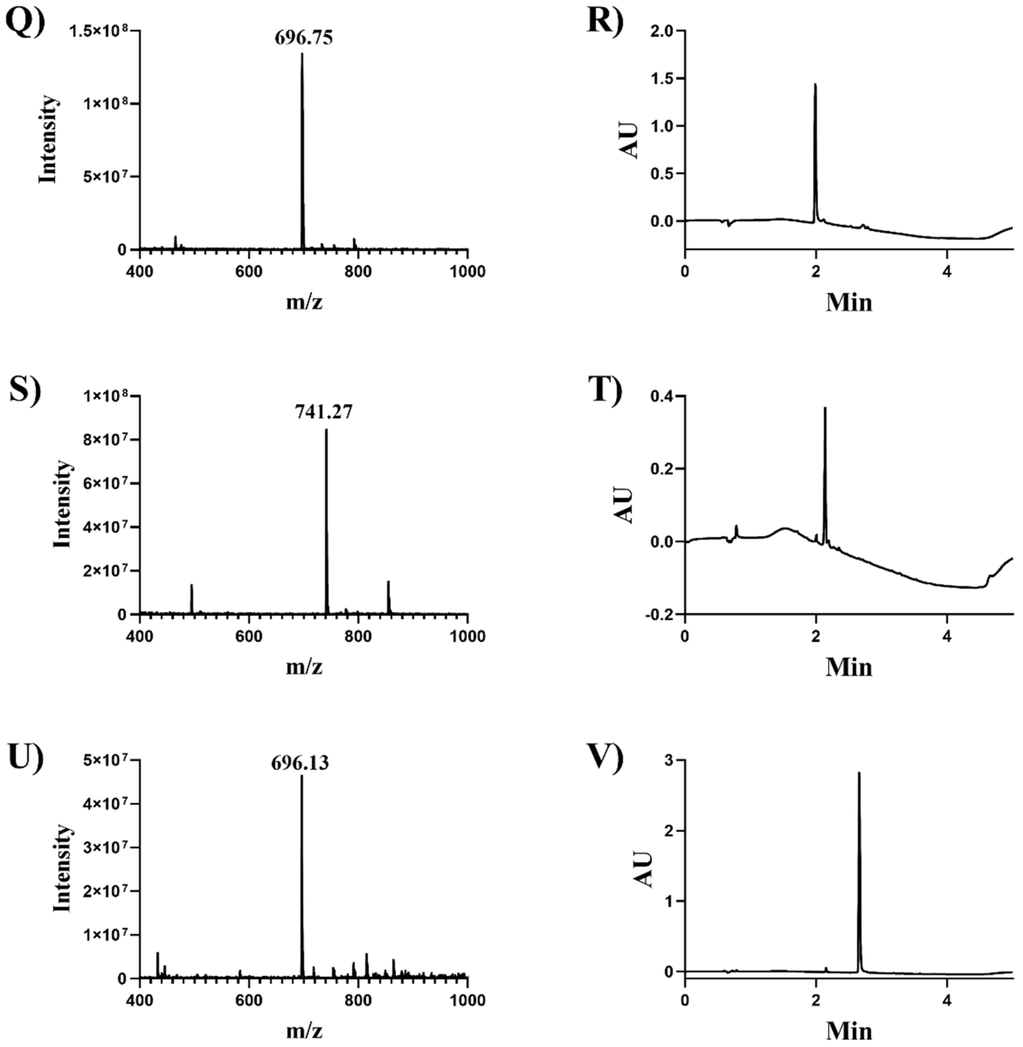

Figure S3 Mass spectra and UPLC of 11 cyclic hexapeptides after cyclization (UV detection at 214 nm). **(A)** The mass spectra of Cy-f1. **(B)** The UPLC profile of Cy-f1. **(C)** The mass spectra of Cy-f2. **(D)** The UPLC profile of Cy-f2. **(E)** The mass spectra of Cy-f3. **(F)** The UPLC profile of Cy-f3. **(G)** The mass spectra of Cy-f4. **(H)** The UPLC profile of Cy-f4. **(I)** The mass spectra of Cy-f5. **(J)** The UPLC profile of Cy-f5. **(K)** The mass spectra of Cy-f6. **(L)** The UPLC profile of Cy-f6. **(M)** The mass spectra of Cy-f7. **(N)** The UPLC profile of Cy-f7. **(O)** The mass spectra of Cy-f8. **(P)** The UPLC profile of Cy-f8. **(Q)** The mass spectra of Cy-f9. **(R)** The UPLC profile of Cy-f9. **(S)** The mass spectra of Cy-f10. **(T)** The UPLC profile of Cy-f10. **(U)** The mass spectra of Cy-f11. **(V)** The UPLC profile of Cy-f11.

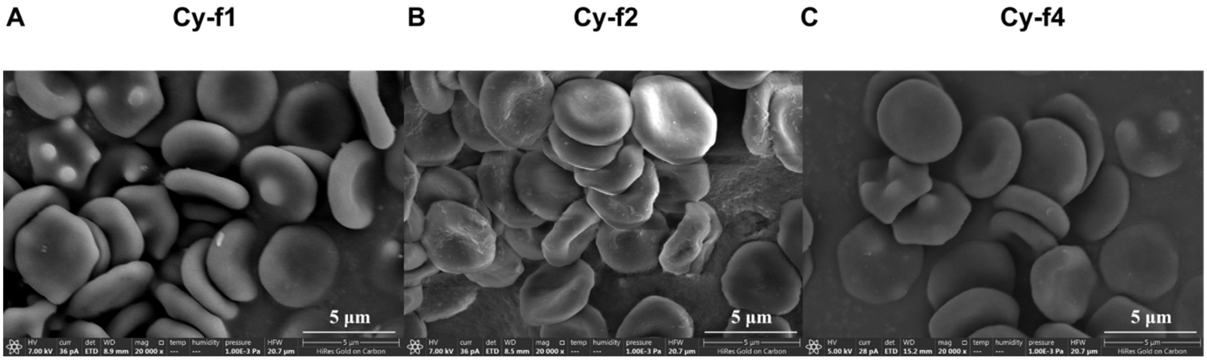

Figure S4. SEM images of RBCs treated with Cy-f1, Cy-f2, and Cy-f4. (A) SEM images of RBCs treated with Cy-f1. (B) SEM images of RBCs treated with Cy-f2. (C) SEM images of RBCs treated with Cy-f4.
